# Invisible Effect of Virtual Water Transfer on Water Quantity Conflict in Transboundary Rivers—Taking Ili River as a Case

**DOI:** 10.3390/ijerph19158917

**Published:** 2022-07-22

**Authors:** Xia Xu, Fengping Wu, Qianwen Yu, Xiangnan Chen, Yue Zhao

**Affiliations:** 1 Architectural Engineering School, Tongling University, Tongling 244000, China; 027627@tlu.edu.cn; 2 Business School, Hohai University, Nanjing 211100, China; 180213120002@hhu.edu.cn (X.C.); zh_yyyyy@hhu.edu.cn (Y.Z.); 3 Business School, Suzhou University of Science and Technology, Suzhou 215009, China

**Keywords:** transboundary river, water stress, virtual water transfer, water quantity conflict

## Abstract

Water stress in countries within a drainage basin exacerbates the water quantity conflict in transboundary rivers. However, few studies considered the invisible effect of virtual water transfer on water quantity conflict by intensifying water stress. Therefore, this study, with Ili River as the case, collects data on Virtual Water Trade (VWT) from 1990 to 2015, uses water stress index (WSI) to assess water stress values under two scenarios (with or without virtual water transfer), and takes Grey Verhulst Model to predict two scenarios water stress values respectively. Next, based on the Levenberg—Marquardt (LM) Algorithm, this study compares the water quantity conflict intensity of the two scenarios, and further explores the invisible effect of virtual water transfer on the conflicts among transboundary rivers. Results show: (1) During the study period (1990–2015), water stress in China and Kazakhstan along the banks of Ili River increased in general. (2) China was basically a net exporter of virtual water during 1990–1995, and Kazakhstan became a net exporter after 1995. (3) During 2020–2025, water conflict value of Ili River without virtual water transfer is 0.458, while the value rises to 0.622 with virtual water transfer, indicating that virtual water transfer between China and Kazakhstan has an invisible enhancement on the water quantity conflict of Ili River. (4) The intensified water quantity conflict is mainly caused by the more and more serious water stress in Kazakhstan. On such basis, it is more urgent for Kazakhstan to restructure its economy and trade.

## 1. Introduction

In the past few decades, social development and water environment have experienced huge changes, thus elevating water stress. It causes countries within a drainage basin to suffer from constant water quantity conflicts [1]. If a country overuses physical water, it would exacerbate water stress in other countries within the same drainage basin, forcing them to fight for common water resources—water quantity conflict. For instance, the supply of renewable water resources in transboundary rivers such as Rio Grande, Colorado River, and Ili River is lower than the total water demand, resulting in much higher water stress and water quantity conflict along the banks [2]. In addition, Syria also has a conflict with neighboring countries over water quantity due to water stress [3,4]. Many scholars believe that any scarce resource would cause conflicts; water resources are no exception [5,6,7,8,9]. Scholars also found that global competition for water resources would “inevitably” intensify, and by 2050, increasingly growing demand for water in regions (except most water-rich ones) may lead to more fierce competition [6,7,10]. In addition, scholars argue that though water quantity conflict in transboundary rivers is influenced by factors such as political and legal agreements, the major factor is still water stress. To be specific, political and legal agreements are based on the independent variable—physical water stress [11,12,13,14,15,16]. It means water stress is a key reason for water quantity conflict.

To analyze the critical relationship between water stress and water quantity conflict in transboundary rivers, Gunasekara et al. [13] established a relational model between the regional vulnerability of water resources and the risk of water quantity conflict. Vesco et al. [17] applied meta-analysis on the relationship between water stress and the incidence of water quantity conflict. Moreover, Gain [18] used integrated evaluation method (including the construction of complex social-ecological system, dynamic assessment systems and participatory approach) to look into the scarcity risks in the Yarlung Tsangpo River. Of which, the participatory approach was used (limited number of stakeholders identify the most relevant issues with reference to water stress risks) to look into the relationship between the conflict and the water stress risk. Gain found that the risk of water shortage includes supply (such as snow melting, monsoon, and sea level rise) and demand (such as population pressure, economic development, and urbanization trends) factors, and as the risk intensifies, conflict may fluctuate with it. These studies help us make clear of the relationship between water stress and water quantity conflict in transboundary rivers. However, they are based on physical water.

Actually, virtual water is interrelated with physical water. Physical water, though mostly consumed during commodity production, would be partially converted into virtual water, then further transmitted as water resources to other countries through international trade [19]. A country might wish to import products that require a lot of water in their production and export products or services that require less water. This implies net import (export) of “virtual water” can reduce (increase) the country’s own physical total water use, invisibly increasing it physical water available, and then will relieve the pressure on the nation’s own water resources [20]. Many scholars believe that virtual water can alleviate the stress on physical water of net import countries, while it is quite the opposite for net export countries [19,20,21,22,23,24,25,26,27,28]. In other words, virtual water trade (VWT) can alter physical water stress in cities within a drainage basin. This transferred water, if included into the available water resources in the riparian areas, would affect physical water stress, yielding an invisible effect on water quantity conflicts in transboundary rivers. Angelis et al. [29] found a correlation between virtual water transfer and water conflict among countries within a drainage basin, and water make the same contributions as petroleum and natural gas do to inter-state conflict. Tian and Wang [30] prove that trade of commodities in countries along transboundary rivers would cause more water transfer, affecting the actual amount of water of these countries, leading to inevitable water conflict. These studies show that virtual water transfer would affect the scarcity of physical water, and further cause water quantity conflict. However, they do not convert or integrate virtual water transfer into water stress index, to quantitively analyze the invisible influence of virtual water transfer on the water quantity water of transboundary rivers. Therefore, the purpose of this study is to measure the virtual water transfer among countries within a drainage basin, and quantify the water quantity conflict, so as to construct a model to assess and predict water stress with or without virtual water transfer. On such basis, this study aims to further construct the mathematical relationship between water stress and water quantity conflict, predict how much water stress would change in the future, and how much water quantity conflict is triggered by such change, in order to quantify the invisible influence aforesaid.

Moreover, in order to achieve the aims in this study, we need to construct the following models. First, the virtual water measurement method. Many scholars found the multi-regional input–output approach can analyze the dependencies between industries and production chains between countries or regions [31,32,33,34,35,36,37,38,39,40]. It provides a clearer quantification of the amount of water deployed in trade and makes the virtual water calculation more intuitive and accurate [31,32,33,34,35,36,37,38,39,40]. Therefore, this study chooses the multi-regional input–output approach to measure virtual water transfer contents. Second, the water stress assessment measurement method. As current water stress assessment indexes (such as Falkenmark Index, Criticality ratio, Water Stress Index, Water Exploitation Index) are all centered on water consumption and water availability [21,41,42,43,44,45,46,47,48,49,50,51,52,53,54]. In addition, we focus on the water stress caused by the consumptive use and water availability of surface water resources. Therefore, this study draws on the water stress index (WSI) and uses ratio of water consumption to water availability, to measure water stress. Third, the water stress prediction measurement method. Some scholars found Grey Verhulst Model handling techniques enable extraction of valid information from known data, and can accurately predict the characteristics of uncertain systems [55,56,57]. Therefore, it can effectively solve the relationship between data with poor information characteristics. Moreover, its processing techniques make it possible to predict data with S-curve growth characteristics more accurately [55,56,57]. S-curve means that things cannot grow indefinitely just like J-curve. When they reach the maximum values, they will stop growing [57]. It is in line with the features of water stress data. Meanwhile, water stress data for transboundary rivers are scarce. Therefore, this study selects the Grey Verhulst Model to predict water stress. Next, methods to quantify water quantity conflict. Currently, scholars measure water quantity conflict with either the number of conflicts (CN) or conflict level (CL), but not a combination of them [13,58]. Unlike previous research, this study combines both CN and CL to determine the intensity of water quantity conflict (CQ). Finally, building the quantitative relation between water stress and water quantity conflict. Currently, the main models for matching mathematical relationships mainly include Genetic Algorithm, Gauss–Newton method, the LM Algorithm. Compared to Genetic Algorithm and Gauss–Newton method, the LM Algorithm fitting mathematical relationships process is able to deal with large numbers of parameters and variables [59]. It is suitable for curve fitting of any non-linearity between variables. On such basis, this study chooses the LM Algorithm to fit the quantitative relation between water stress and water quantity conflict.

Based on the above analysis, there is no quantitative study on the invisible effect of virtual water transfer on water quantity conflict by intensifying water stress. In order to fill the gap, this study aims to: (1) Measure virtual water transfer in countries within a drainage basin, then integrate it into the WSI. (2) Determine indicators and models to measure water stress under two scenarios (with or without virtual water transfer). (3) Quantify the water quantity conflict in transboundary rivers, and construct the relational model between water stress and water quantity conflict under the two scenarios.

This paper mainly makes the following contributions: (1) Revealing the invisible effect of virtual water transfer on water quantity conflict in transboundary rivers, to help countries within the same drainage basin to formulate fair and reasonable strategies to relieve water quantity conflict. (2) Integrating virtual water transfer into water stress index to make assessment more accurate.

The rest of this paper is structured as follows: Section 2 describes research framework and study area; Section 3 introduces models; Section 4 is the major results; Section 5 is the discussion; and Section 6 presents the conclusion.

## 2. Research Framework and Study Area

### 2.1. Research Framework

**Figure 1:**
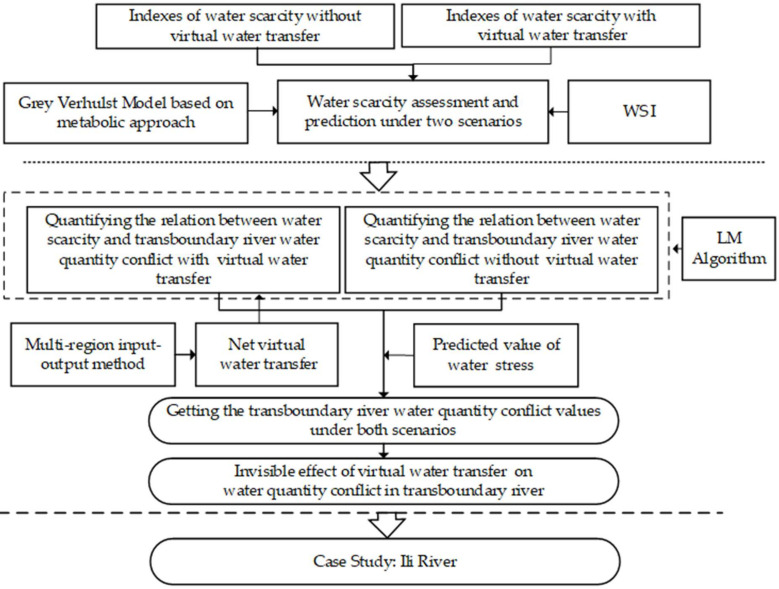
Research framework.

First, this study selects indexes of water stress under two scenarios, and constructs corresponding models to assess and predict water stress. Second, virtual water transfer is measured, then converted pro rata into WSI under virtual water transfer. Next, the quantitative relation between water stress and water quantity conflict under both scenarios is constructed. Finally, the predicted WSI is put into the corresponding relations, to obtain and compare the predicted CQ, so as to measure the invisible effect of virtual water transfer on water quantity conflict in transboundary rivers (Figure 1).

Moreover, WSI original definition is a ratio between water consumption and water availability intended to measure water stress in a country [21,41,42,43,44,45,46,47,48,49,50,51,52,53,54]. It does not care if the products and services are for exporting and does not include goods and services consumed in the country with water abstracted in other countries [21,41,42,43,44,45,46,47,48,49,50,51,52,53,54]. In this paper, virtual water is embedded in the water stress indicators. At the national level, in principle, when the available water resources of country R decreases by X, the available water resources of country S increases by X. Moreover, the water consumed in country R to produce goods and services for exporting (X) is deducted from the total water consumption of country R. The water consumed in country S must be increases by X. While, at the basin level, after basin countries trade generated virtual water transfer, it is redistributed within the country. Consequently, it triggers water availability changed in the coastal zone of each riparian country. It means that the invisible effect of virtual water trade can be characterized by the water availability indicators change. Furthermore, actually, at the basin level, as the amount of virtual water trade between regions is lower and trade data are more difficult to obtain, the effect of virtual water trade between riparian regions on the indicator is not considered in the water consumption.

### 2.2. Study Area

#### 2.2.1. Overview

(1) Brief introduction of Ili River

There are 24 transboundary rivers between China and Kazakhstan, of which Ili River is a critical one with obvious water quantity conflict. So, this study chooses the Ili River as the case. Ili River is located in the Ili-Balkhash basin, with temperate continental climate, suffering from drought and water shortage. The Ili River basin covers an area of 151.2 × 10^3^ km^2^, of which 94.5 × 10^3^ km^2^ is in Kazakhstan and 56.7 × 10^3^ km^2^ in Xinjiang, China. The total volume of surface runoff of the Ili River basin is 228.7 × 10^8^ m^3^, of which 170.4 × 10^8^ m^3^ (about 74.5%), is produced in Xinjiang, and 58.3 × 10^8^ m^3^ (about 25.5%) in Kazakhstan [60]. While the annual net flow of the Ili River basin in China to Kazakhstan is 130 × 10^8^ m^3^ per year [60]. So, Xinjiang makes more contributions in producing runoff yet uses less fresh water.

(2) Overview of water quantity conflict

Ili River flows from East in Xinjiang, China to West in Eastern Kazakhstan and the middle and lower courses are in Kazakhstan—both are severely arid areas. As economy advances and population increases, consumptive water use (mainly for agriculture) of Ili River rockets [61]. The inherent water stress causes water quantity conflict between China and Kazakhstan. Moreover, the disagreement about the allocation of water rights in the Ili River between the two countries has made it difficult to quell the water conflict. For a long time, Xinjiang’s increase in water use has raised Kazakhstan’s concerns about its water use security. Any fluctuation in the water quantity out of Xinjiang would cause unsatisfaction from Kazakhstan. China asserts that Xinjiang uses much less water than Kazakhstan yet makes greater contributions to the net runoff of the Ili River. Therefore, water quantity conflict between the two countries is centered around who overuses more water of the Ili River [60]. Since the 1990s, China and Kazakhstan have been negotiating on relevant issues of transboundary rivers and great achievements have been made. However, they have not come to an agreement on the fair use of water of the Ili River basin.

#### 2.2.2. Data Sources

Data on virtual water transfer are collected from the EORA website [62]. Five Global Input-Output Tables are available for academic use: World Input-Output Database (WIOD), Inter-Country Input-Output Tables (ICIOT), EXIOBASE, Global Trade Analysis Project (GATP), and EORA. The public data between China and Kazakhstan during 1990–2015 is only available from EORA. Therefore, we chose it.

To get a more accurate and scientific result, the water stress data of Ili River from 1990 to 2015 is chosen accordingly. Water stress data—annual rainfall, annual evaporation, water consumption (agricultural, industry, domestical use)—is from academic papers, NASA and ESA databases. Further calculations are performed based on such data [63,64,65,66]. Moreover, the Ili River’s water quantity conflict data between China and Kazakhstan during 1990–2015 is collected from the official website of Ministry of Ecology and Environment of People’s Republic of China and Transboundary Freshwater Resources Disputes (TFDD) [58,67].

## 3. Model

The main purpose of the model is to taking the predicted water stress index values into the mathematical relation between water quantity conflict and water stress, and measuring the water quantity conflict values of the two scenarios (with or without virtual water transfer). Then revealing the invisible effect of virtual water transfer on water quantity conflict. To obtain the objective, the model is divided into three main parts.

The first part is to measure the virtual water transfer. The second part is to determine the WSI for both scenarios (with or without virtual water transfer), and to construct predictive model for predicting WSI respectively. The third part is to determine the water quantity conflict, and then to construct a relationship between the WSI (from the second part) and the water quantity conflict in transboundary rivers. Meanwhile, the predicted WSI (from the second part) is put into the corresponding relations, to obtain and compare the predicted water quantity conflict, so as to measure the invisible effect of virtual water transfer on water quantity conflict.

### 3.1. Virtual Water Transfer Model

There are both bottom-up [68] and top-down approaches [69] for building the virtual water measurement model, and the top-down input–output approach produces more visualized and accurate results [31,32,33,34,35,36,37,38,39,40,70]. Therefore, this study chooses the multi-regional input–output approach to measure virtual water transfer.

First, assume that a transboundary river crosses (m − 1) countries within a drainage basin, and denote them as country 1, country 2, … and country m − 1. Other countries outside the basin are denoted as other country m. Second, based on the multi-regional input–output table, a modified multi-regional input–output table is constructed (See Table A1 in Appendix B).

Based on the multi-regional input–output approach (Table A1), virtual water transfer between two countries is obtained as follows:

(1) $$Z^{rs}=\overset{m}{\underset{p=1}{\sum }}W^{r}L^{rp}F^{ps}\;$$

where r and s refer to countries within a drainage basin, p stands for countries trading with country r and country s, m is the number of countries trading with country r and country s, and $Z^{rs}$ is the virtual water transfer from country r to country s. When p = r, $Z^{rs}$ is the virtual water transfer from direct trade from country r to country s. When p≠r, $Z^{rs}$ is the indirect virtual water transfer from country r to country s—country r exports intermediate products to country p, which are then processed by country p into final products and exported to country s, thus transferring water embedded in products from country r to country s.

W refers to the direct water coefficient matrix. $W^{r}$ is the direct water coefficient matrix of country r, representing the direct water use per unit of output in each sector of country r. L stands for the Leontief Inverse Matrix, $L^{rp}$ is the submatrix of L from country r to country p, standing for the total production that each sector generates of country r to satisfy the country p final demand of the economy [71,72]. F is the final demand vector, and $F^{ps}$ is the submatrix of the final demand matrix from country p to country s, representing the final demand in each industry sector of country s comes from each industry sector of country p [71,72].

Similarly, the virtual water transfer from country s to country r is as follows:

(2) $$Z^{sr}=\overset{m}{\underset{p=1}{\sum }}W^{s}L^{sp}F^{pr}\;$$

where $W^{s}$ stands for the direct water coefficient matrix of country s, $L^{sp}$ is the submatrix of L from country s to country p, $F^{pr}$ is the submatrix of the final demand matrix from country p to country r.

Therefore, the net virtual water transfer from country r to country s is as follows:

(3) $$kz^{rs}=\overset{m}{\underset{p=1}{\sum }}W^{r}L^{rp}F^{ps}-\overset{m}{\underset{p=1}{\sum }}W^{s}L^{sp}F^{pr}\;$$

where $kz^{rs}$ is the net virtual water transfer from country r to country s.

Likewise, the net virtual water transfer from country s to country r is as follows:

(4) $$kz^{sr}=\overset{m}{\underset{p=1}{\sum }}W^{s}L^{sp}F^{pr}-\overset{m}{\underset{p=1}{\sum }}W^{r}L^{rp}F^{ps}\;$$

### 3.2. Model of Water Stress

Section 3.2 focuses on the building water stress model for the coastal zone of the transboundary river with or without virtual trade. It involves two main steps. Step 1: Determining water stress values at the basin riparian zone level. Moreover, given that the trade between countries within the basin is difficult to measure and relatively small, these indexes do not take into account the virtual water transfers. Namely, the data for these basin-level water stress indicators (indicators on numerator) are the same. Step 2: After basin countries trade generated virtual water transfer, it is redistributed within the country. Consequently, it triggers water availability change in the coastal zone of each riparian country, converting the virtual water transfers data at the national level to match the water stress indicators at the basin level. Then the virtual water transfer is used as a stand-alone increase indicator of water stress for the riparian zone of the basin, and embedded in the water availability.

The above two steps make us to construct water stress models for the basin coastal zone under two scenarios respectively.

#### 3.2.1. Indicators and Assessment Model to Assess Water Stress without Virtual Water Transfer

(1) Indicators to assess water stress without virtual water transfer

Water stress assessment models mainly include Falkenmark Index (Per capita water availability, FI) [42], Water Stress Index (the ratio of total annual freshwater withdrawals to annual water availability, WSI) [48], Criticality Ratio (Ratio of water use to availability, CR) [42], and Water Exploitation Index (the ratio of abstraction minus returns to renewable water resources minus environmental flow) [43,44,45]. Although there are differences in the literal meaning of these definitions, actually, the essence of these definitions all centered on water consumption and water availability. Namely, they are the same.

Therefore, this study draws on the water stress index (WSI) and uses ratio of water consumption to water availability, to measure water stress. Annual water consumption includes water for industrial, agricultural, and domestic use [21,46,48]. Annual water availability (annual runoff) equals annual precipitation minus annual evapotranspiration. Moreover, given that the ecosystem also needs water to provide goods and services for human beings, this study takes ecological water demand into consideration. In addition, desalinated water and imported physical water are also included as water availability in this study. Indicators to assess water stress without virtual water transfer are detailed in Table 1.

(2) Water stress assessment model without virtual water transfer

a. Formula to assess water stress

Based on the data in Table 1 and the model constructed by some scholars [21,46,48], this study constructs a model to assess water stress of the riparian area of transboundary river in country *r* without virtual water transfer. The formula is as follows:

(5) $$X^{r}=\frac{\sum_{h=1}^{4}W_{h}^{r}}{P^{r}-ET^{r}+Q_{3}^{r}+Q_{4}^{r}}\;\left(r=1,2,3,\ldots ,m-1\right)$$

where r means riparian zone of transboundary river in country r. X means WSI. $X^{r}$ is the WSI of riparian areas in country r, $W_{h}^{r}\left(h=1,2,3,4\right)$ refers to the total annual water use, and $P^{r}-ET^{r}+Q_{3}^{r}+Q_{4}^{r}$ is the total annual water availability. The specific calculation process of water stress indicators is shown in the Appendix A.

b. Water stress prediction model

In order to predict the impact of water stress of riparian zone on transboundary river water quantity conflict, we need to predict water stress values.

**Figure 2:**
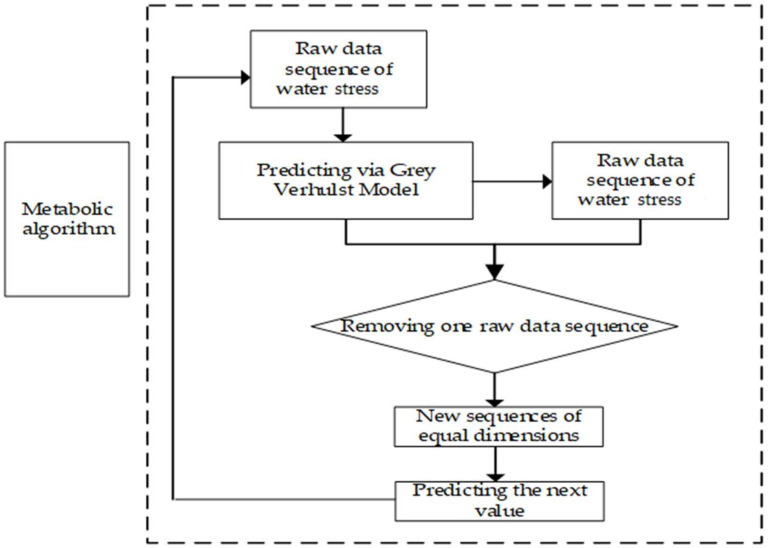
Grey Verhulst Model based on metabolic approach.

Current forecasting methods include Gray Verhulst Model, Interpolation method, Time Series Forecasting methods etc. Based on the continuous regularity in the development of objective things, time series forecasting method uses historical data from the past to speculate on future trends through statistical analysis [73]. Interpolation prediction methods focus on predicting images related to the trajectory of an object’s movement [74]. Although, they require sufficient historical real data. Grey Verhulst Model can effectively solve the relationship between data with poor information characteristics [55,56,57]. Moreover, its processing techniques make it possible to predict data with S-curve growth characteristics more accurately [55,56,57]. It is in line with the features of water stress data. Meanwhile, water stress data for transboundary rivers are relatively difficult to obtain. Moreover, the metabolic approach is to replace the oldest original data one by one, which is more compatible with the current situation, and can reduce the error [56]. Therefore, the Gray Verhulst Model based on metabolic approach is applied to predict water stress. Details are shown in Figure 2 and Formulas (6)–(18).

Let $X^{\left(0\right)}=\left(x^{\left(0\right)}\left(1\right),x^{\left(0\right)}\left(2\right),\cdots ,x^{\left(0\right)}\left(k\right)\right)$ be the data sequence, with $x^{\left(0\right)}\left(k\right)>0$ and $X^{1}\left(t\right)$ as the cumulative sequence (1 − AGO) of $X^{0}\left(t\right)$:

(6) $$X^{\left(1\right)}=\left(x^{\left(1\right)}\left(1\right),x^{\left(1\right)}\left(2\right),\cdots x^{\left(1\right)}\left(k\right)\right)$$

where

(7) $$x^{\left(1\right)}\left(k\right)=\overset{k}{\underset{g=1}{\sum }}x^{\left(0\right)}\left(g\right),g=1,2,\ldots k$$

(8) $$x^{\left(0\right)}\left(k\right)+b\pi^{\left(1\right)}\left(k\right)=c{\left(\pi^{\left(1\right)}\left(k\right)\right)}^{\alpha }$$

Formula (8) is Gray Model GM (1,1).

where

(9) $$\pi^{\left(1\right)}\left(k\right)=\frac{1}{2}\left(x^{\left(1\right)}\left(k\right)+x^{\left(1\right)}\left(k-1\right)\right)$$

When α = 2, Formula (8) becomes

(10) $$x^{\left(0\right)}\left(k\right)+b\pi^{\left(1\right)}\left(k\right)=c{\left(\pi^{\left(1\right)}\left(k\right)\right)}^{2}$$

Formula (10) is Gray Verhulst Model. The result of whitening Formula (10) is:

(11) dx1dt+bx1=cx12

The solution to Formula (10) is:

(12) x1t=1ebt1x10−cb1−e−bt=bx10cx10+b−cx10ebt 

The time response equation of Formula (10) is:

(13) x^1k+1=bx10cx10+b−cx10ebk

According to the metabolic method, original data, starting the first one, are replaced one by one, so as to minimize errors, and achieve medium and long-term prediction.

The Least Squares Estimation of the Grey Verhulst Model parameter list $\hat{b}={\left[b,c\right]}^{T}$ is

(14) $$\hat{b}={\left(B^{T}B\right)}^{-1}B^{T}Y$$

(15) $$B=\left|\begin{array}{cc} -\pi^{\left(1\right)}\left(2\right) & {\left(\pi^{\left(1\right)}\left(2\right)\right)}^{2}\\ -\pi^{\left(1\right)}\left(3\right) & {\left(\pi^{\left(1\right)}\left(3\right)\right)}^{2}\\ \vdots & \vdots \\ -\pi^{\left(1\right)}\left(k\right) & {\left(\pi^{\left(1\right)}\left(k\right)\right)}^{2} \end{array}\right|,\;Y=\left|\begin{array}{c} x^{\left(0\right)}\left(2\right)\\ x^{\left(0\right)}\left(3\right)\\ \vdots \\ x^{\left(0\right)}\left(k\right) \end{array}\right|$$

Hence, based on Formula (13), water stress data for year *k* + 1 are:

(16) $${\hat{x}}^{\left(0\right)}\left(k+1\right)={\hat{x}}^{\left(1\right)}\left(k+1\right)-{\hat{x}}^{\left(1\right)}\left(k\right)\;$$

where ${\hat{x}}^{\left(0\right)}\left(k\right)$ is the original data simulation sequence.

Moreover, error test for Grey Verhulst Model is as follows:

(17) $$\varepsilon^{\left(0\right)}\left(k\right)=x^{\left(0\right)}\left(k\right)-{\hat{x}}^{\left(0\right)}\left(k\right)\;$$

where $\varepsilon^{\left(0\right)}\left(k\right)$ is the residual sequence.

(18) $$\Delta_{k}=\left|\frac{\varepsilon \left(k\right)}{x^{\left(0\right)}\left(k\right)}\;\right|\;\left(18\right)\;$$

$\Delta_{k}$ is the simulated relative error at point *k*, and $\bar{\Delta }=\frac{1}{k}\overset{k}{\underset{g=1}{\sum }}\Delta_{k}$ is the mean relative error. With a given γ (usually as 0.1 [55]), when $\bar{\Delta }<\gamma$ and $\Delta_{k\;}<\gamma$, the model is the qualified residual-test model.

#### 3.2.2. Indicators and Assessment Model to Assess Water Stress with Virtual Water Transfer

(1) Indicators to assess water stress with virtual water transfer

Through the Section 2.1 analysis, under virtual water transfer, only the indicator of water availability added virtual water transfer, and the indicator of water consumption is not changed. The indicator water availability to assess water stress includes rainfall, evapotranspiration, imported physical water, and desalinated water as well as virtual water transfer in international trade. The indicators are listed in Table 2.

(2) Formula of water stress with virtual water transfer

a. Formula to assess water stress

Based on Table 2, this study constructs a water stress assessment model with virtual water transfer. The formula is as follows:

(19) $$X^{r\prime }=\frac{\sum_{h=1}^{4}W_{h}^{r}}{P^{r}-ET^{r}+Q_{3}^{r}+Q_{4}^{r}+Q_{5}^{r}}\;\left(r=1,2,3,\ldots ,m-1\right)$$

where $X^{r\prime }$ is WSI of country r after integrating virtual water transfer. Given that the trade between countries within the basin is difficult to measure and relatively small, except $Q_{5}^{r}$, other indicators do not take into account the impact of virtual water trade.

In addition, by converting and integrating virtual water transfer into the water availability of riparian areas of countries within the same drainage basin, $Q_{5}^{r}$ can be obtained:

(20) $$Q_{5}^{r}=\Gamma \times \left(-kz^{rs}\;\right)$$

where Γ is the discount ratio, and Γ>0. Since input–output data for the riparian zone of a basin are difficult to obtain, and countries within a drainage basin may share dozens of transboundary rivers, this study scales down the virtual water at national level and apportions to each transboundary river pro rata. $kz^{rs}$ is the net volume of virtual water transfer of country r to s. If country *r* is a net importer, means $Z^{sr}$ is greater than $Z^{rs}$, $Q_{5}^{r}>0$, and its total water availability increases; if country r is a net exporter, $Z^{sr}$ is less than $Z^{rs}$, $Q_{5}^{r}<0$, and its total water availability decreases; if $Q_{5}^{r}=0$, $Z^{sr}$ is equal to $Z^{rs}$, both sides of the trade do not affect each other’s water resources, and the total water availability of country r remains unchanged, or the two countries do not trade at all.

b. Predicted WSI

Similarly, after assessing water stress with virtual water transfer, this study integrates it into the Grey Verhulst Model based on metabolic method, to obtain the predicted WSI with virtual water transfer.

### 3.3. Constructing the Mathematical Relation between Water Stress and Water Quantity Conflict

In order to determine the mathematical relationship between water stress and water quantity conflict, we need to base on CL and CN to determine CQ. See Appendix A for the detailed calculation process.

About the model of the quantitative relationship between water stress and water quantity conflict, Genetic Algorithm, Gauss–Newton method, the LM Algorithm all can fit the mathematical relation of variables [59]. Although, Genetic Algorithm can only deal with general non-linear parameter in fitting processes [59]. Gauss–Newton algorithm can fit complicated non-linear equations, but the initial values must be set [59]. The LM Algorithm is development from Gauss–Newton, while it does not need to set an initial value. Therefore, it solves the problem that if the initial value is not set correctly, the parameter solution does not gather during the iterative process and thus the optimal solution cannot be found [59]. By comparison, we find that the LM Algorithm is superior in the mathematical relationship fitting process. Therefore, in this paper, the LM Algorithm is chosen to construct the mathematical relation between water stress and water quantity conflict.

With current CQ in transboundary rivers and WSI determined, the mathematical relation between water quantity conflict and water stress is then repeatedly optimized via the LM Algorithm and the 1stOpt software. It has been created by Seven Dimensions High Technology Corporation (located in Beijing, China)

(1) Mathematical relation without virtual water transfer

Let CQ and WSI ($x_{1},\;x_{2},\cdots and\;x_{m-1})$ meet the following relationship:

(21) $$\mathrm{CQ}=f\left(x_{1},\;x_{2},\cdots ,x_{m-1};d_{1},\;d_{2},\cdots ,d_{p}\right)+\sigma$$

where *f* is the nonlinear function of undetermined parameters—$d_{1},\;d_{1},\cdots ,d_{p}$, σ is the error term between the estimated and actual output values. See Appendix A for detailed solution procedures.

(2) Mathematical relation with virtual water transfer

Similarly, after integrating virtual water, water quantity conflict (CQ′) has the following correlation with water stress index ($x_{1}^{\prime },\;x_{2}^{\prime },\cdots ,x_{m-1}^{\prime }$):

(22) $$\mathrm{CQ}\prime =f\left(x_{1}^{\prime },\;x_{2}^{\prime },\cdots ,x_{m-1}^{\prime };d_{1}^{\prime },\;d_{2}^{\prime },\cdots ,d_{p}^{\prime }\right)+\sigma^{\prime }$$

where *f* is the nonlinear function undetermined parameters $d_{1}^{\prime },\;d_{2}^{\prime },\cdots ,d_{p}^{\prime }$*,* $\sigma^{\prime }$ is the error term between the estimated and actual output values after integrating virtual water, and the solution procedures are the same with those of no virtual water transfer.

## 4. Results

### 4.1. Net Virtual Water Transfer between China and Kazakhstan

**Figure 3:**
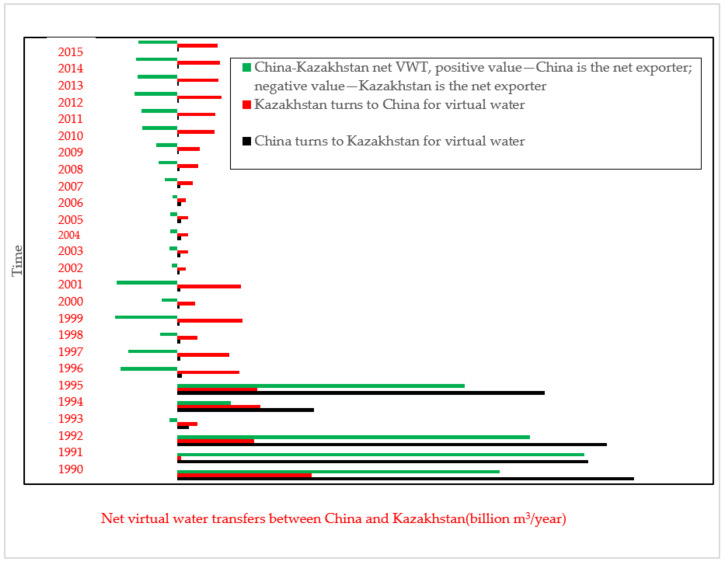
1990–2015 Comparison of virtual water transfer between China and Kazakhstan.

This study collects data on virtual water transfer between China and Kazakhstan during 1990–2015 from Eora (see Figure 3). In general, during 1990–1995, China was a net exporter and transferred more virtual water to Kazakhstan, with the output reaching the peak of 42.11 billion m^3^ in 1991. After 1995, Kazakhstan became a net exporter, with the highest net output reaching 8.16 billion m^3^ in 1999.

### 4.2. Assessment and the Predicted WSI under Both Scenarios

#### 4.2.1. The Predicted WSI without Virtual Water Transfer

(1) Changes in the total water consumption of the Ili River basin

**Figure 4:**
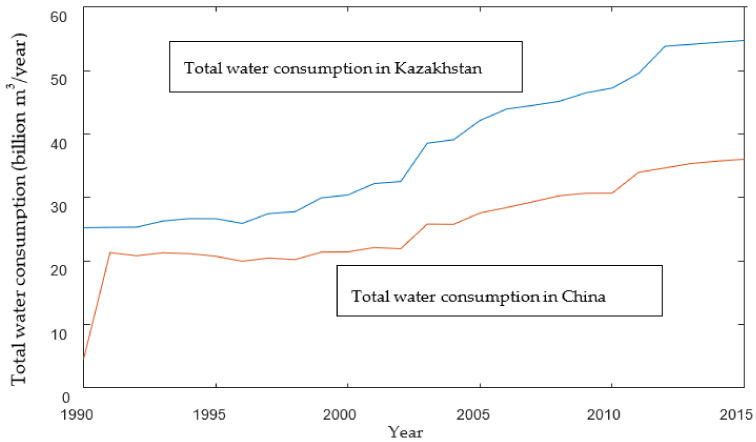
Total water consumption in the study area without virtual water transfer.

Changes in the total water consumption of the study area from 1990 to 2015 are shown in Figure 4.

According to Figure 4, the total water consumption from 1990 to 2015 of the two countries showed an overall upward trend and Kazakhstan consumed more water than China. Specifically, the total water consumption of the two countries increased slowly from 1990 to 2000. The water consumption in China increased from 2.1345 billion m^3^ in 1990 to 2.1432 billion m^3^ in 2000 by about 0.4%, and in Kazakhstan from 2.5284 billion m^3^ in 1990 to 3.0401 billion m^3^ in 2000 by about 16%. The percentage rose up to 27.8% in China and 31.9% in Kazakhstan in 2010. After that, both China and Kazakhstan were aware of the importance of environmental protection and committed to sustainable development, so the percentage dropped to 5.7% and 9.5% respectively.

(2) Changes in the total water availability of the study area

**Figure 5:**
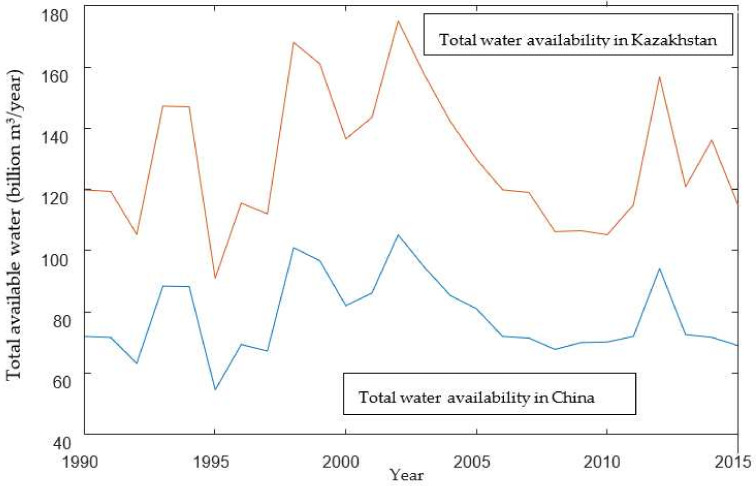
Total water availability in the study area without virtual water transfer.

According to Figure 5, the water availability in the Ili River fluctuated from 1990 to 2015 in the two countries. The average total water availability over the years in China and Kazakhstan were roughly 81.42 × 10^8^ m^3^ and 134.84 × 10^8^ m^3^ respectively.

(3) Water stress in the study area

To determine the mathematical relationship between water quantity conflict and water stress index values, both of water quantity conflicts and water stress index values are calculated on a five-year cycle. Therefore, based on Formula (5), Figure 4 and Figure 5, we can obtain the WSI during 1990–1995, 1995–2000, 2000–2005, 2005–2010, and 2010–2015 without virtual water transfer. Details of WSI without virtual water transfer are shown in Table 3. In addition, Grey Verhulst Model based on metabolic method is applied to get the WSI of the study area during 2020–2025—0.426 (China) and 0.429 (Kazakhstan) respectively. $\Delta_{k}$ and $\bar{\Delta }$ are below 0.1, in line with the requirement of Grey Verhulst Model on errors, proving that the predicted values are reasonable.

Table 3 shows that water stress showed an overall upward trend, especially after 2005. The main reason is that rapid economic development and a growing population required larger total water consumption [61].

#### 4.2.2. Assessment and the Predicted WSI with Virtual Water Transfer

(1) Estimated water availability in the Ili River basin of China and Kazakhstan

**Figure 6:**
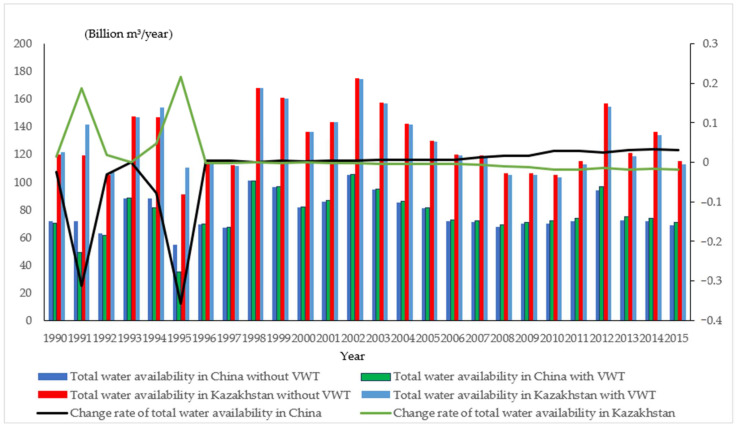
Changes in the total water availability in the study area under both scenarios.

The net transfer of virtual water for between China and Kazakhstan during 1990–2015 is converted (net import = positive, net export = negative), then integrated into the total water availability, to demonstrate the changes, as shown in Figure 6.

According to Figure 6, China had more available water in the riparian zone of Ili River, while Kazakhstan had less. To be more specific, during 1990–1995, the virtual water transfers experienced fluctuations, leading to high change rate in the total water availability. The highest change rates in China and Kazakhstan amounted to 31.2% and 18.7% respectively. Since China was a net exporter, its total water availability dropped, while Kazakhstan was exactly the opposite. After 1995, China became a net importer and Kazakhstan a net exporter, so the total water availability in China increased while that in Kazakhstan relatively decreased. In terms of the change rate, after 1995, the total water availability in both countries stayed relatively stable under both scenarios (with or without virtual water transfer). Meanwhile, in terms of the absolute quantity of total available water, China had relatively less available water to use under both scenarios.

(2) Water stress in the riparian zone of Ili River in two countries

Moreover, according to Figure 6, virtual water transfer exerts an influence on the total water availability in both countries. With Formulas (23) and (24), WSI in the study area from 1990 to 2015 after embedding virtual water is calculated, as shown in Table 4. Similarly, with virtual water transfer, Grey Verhulst Model based on metabolic method is applied to get the WSI of the study area during 2020–2025—0.425 (China) and 0.433 (Kazakhstan) respectively. $\Delta_{k}$ and $\bar{\Delta }$ are below 0.1, in line with the requirement of Grey Verhulst Model on errors, proving that the predicted values are reasonable.

Combining Figure 6 and comparing Table 3 and Table 4, with the addition of virtual water, water stress in both the study area increased in 1990–1995; conversely, water stress in the riparian zone of the Ili River in Kazakhstan increased after 1995.

### 4.3. Mathematical Relation between Water Conflict and Water Stress in Transboundary Rivers under Both Scenarios

(1) Weights of CN, CL and CQ

According to the entropy weight method and other typical transboundary rivers with water quantity conflicts (Table A2), CN ($w_{1})=0.4774$ and CL ($w_{2})=0.5229$ are determined. Accordingly, CQ during 1990–1995, 1995–2000, 2000–2005, 2005–2010, and 2010–2015 are found to be 0.159, 0.174, 0.333, 0.667, and 1.000 respectively.

(2) Mathematical relation between water quantity conflict and water stress without virtual water transfer

This study fits and optimizes the data on water conflict and water stress in the study area via the LM Algorithm and the 1stopt software, and finds their quantitative relation:

(23) $$\mathrm{CQ}=0.4582+0.5451e^{-0.5{\left(\frac{\left(x^{\mathrm{China}}-0.4529\right)}{-0.0057}\right)}^{2}}-0.2994e^{-0.5{\left(\frac{\left(x^{\mathrm{Kazakhstan}}-0.2126\right)}{0.0258}\right)}^{2}}$$

where $x^{\mathrm{China}}$ is WSI in China, and $x^{\mathrm{Kazakhstan}}$ WSI in Kazakhstan. Fitting coefficient R^2^ is close to 0.9, indicating a reasonable functional relation. Next, according to Table 3 and the WSI in China and Kazakhstan during 2020–2025, CQ is found to be 0.458. Without virtual water transfer, the constant variable in the mathematical relationship between water quantity conflict and water stress is 0.4582. The base variable for the effect of water stress in China on water quantity conflict in the Ili River is 0.5451, while the base variable in Kazakhstan is −0.2994.

(3) Mathematical relation between water quantity conflict and water stress with virtual water transfer

Similarly, this study integrates virtual water transfer and establishes the mathematical relation between water conflict and water stress via the LM Algorithm and the 1stOpt software. Fitting coefficient R^2^ is close to 0.8, indicating a sound fit and a reasonable functional relation. Details are as follows.

(24) $$\mathrm{CQ}=0.0712+0.1084e^{-0.5{\left(\frac{\left(x^{\mathrm{China}}-0.2888\right)}{0.0486}\right)}^{2}}+1.4732e^{-0.5{\left(\frac{\left(x^{\mathrm{Kazakhstan}}-0.3714\right)}{0.0588}\right)}^{2}}$$

Next, WSI during 2020–2025 in Table 4 is combined into Formula (22), and results show that after integrating virtual water transfer, CQ of Ili River during 2020–2025 becomes 0.622. With virtual water transfer, the constant variable in the mathematical relationship between water quantity conflict and water stress is 0.0712. The base variable for the effect of water stress in China on water quantity conflict in the Ili River is 0.1084, while the base variable in Kazakhstan is 1.4732.

## 5. Discussion

### 5.1. Analysis on the Changes in Virtual Water Transfer and Water Stress in the Study Area

#### 5.1.1. Analysis on Virtual Water Transfer in China and Kazakhstan

According to Figure 3, Kazakhstan was a net exporter of virtual water since 1995. However, from 1990 to 2000, virtual water transfer between the two countries was unstable, influenced by the political structure and economic transformation of Kazakhstan. In addition, during 1990–2000, both countries mainly consumed water for agriculture. After 2000, their overall virtual water transfer increased; and with the economy of Kazakhstan stabilized and its trade relations with China strengthened, virtual water transfer in Kazakhstan dropped while the total net virtual water export increased.

#### 5.1.2. Analysis on the Invisible Effect of China–Kazakhstan Virtual Water Transfer on Water Stress

(1) Without virtual water transfer

**Figure 7:**
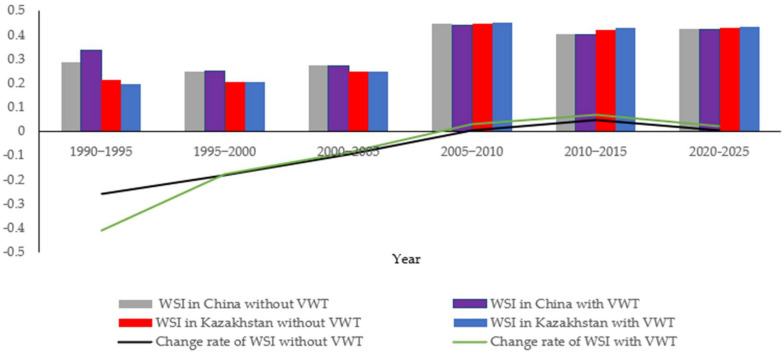
Change rate of WSI in the study area under both scenarios.

According to Figure 4 and Figure 5, water stress during 2010–2015 was lower than that during 2005–2010. Meanwhile, due to the increased total water consumption and the decreased total water availability, WSI in both countries had rocketed since 2005. Moreover, water stress declined during 2010–2015 due to the increase in the total water availability of the Ili River. In the meantime, according to Table 3 and Table 4, WSI during 2021–2025 climbs up (See the gray and red bar lines in Figure 7).

(2) With virtual water transfer

In Figure 7, of all six periods, during the period of 1990–1995, virtual water transfer included in commodity trade between the two countries intensified water stress in China, while things go the other way around in the remaining five periods, including the predicted period of 2020–2025.

(3) Changes in WSI under both scenarios

In Figure 7, change rate of WSI without virtual water transfer increased from −0.259 in 1990–1995 to 0.006 in 2020–2025, and Change rate of WSI with virtual water transfer went up from −0.411 in 1990–1995 to 0.020 in 2020–2025. The result indicates that VWI increased the change rate of water stress. Generally speaking, in every five years, the change rate of WSI with virtual water transfer is greater than that of WSI without virtual water transfer, meaning that the integration of virtual water transfer leads to a wider gap in water stress between China and Kazakhstan.

Water can be redistributed through, in physical terms, water transfer projects and virtually, embodied water for the production of traded products. Such water redistributions can help mitigate or aggravate physical water stress. So, basin states should consider virtual water transfers, and make water rights allocation schemes more equitable and reasonable. In this paper, through virtual water transfer, net exporting countries (Kazakhstan) relieved water stress of net importing countries (China). Therefore, in the course of allocating water rights, net importing countries (China) can combine with the actual conditions to give net exporting countries (Kazakhstan) more room to negotiate.

### 5.2. Analysis on the Invisible Influence of China–Kazakhstan Virtual Water Transfer on the Water Quantity Conflict of Ili River

#### 5.2.1. Analysis on the Causes of China–Kazakhstan Water Conflict over Ili River

According to Formulas (23) and (24), during 2020–2025, CQ between the two countries is 0.458 without virtual water transfer and 0.622 with virtual water transfer, meaning that virtual water transfer has an invisible enhancing effect on the water conflict of Ili River. Moreover, according to the analysis in Section 5.1.2, virtual water transfer leads to an increase in the change rate of water stress in both countries. Therefore, virtual water transfer included in the trade relation between China and Kazakhstan has an invisible enhancing effect on the water conflict of Ili River.

The base variable for the effect of water stress in Kazakhstan on water quantity conflict is  1.4732  (Formula (24)), much higher than 0.1084 in China, which indicates that water conflict in the Ili River is driven by water stress in Kazakhstan. Kazakhstan’s disagreement about the allocation of water rights of Ili River is the main reason for the water conflict between the two countries. Other main reasons include: First, Kazakhstan suffers from water shortage, thus sensitive to water resources; second, Kazakhstan is at the lower stream of Ili River, influenced by any fluctuation in water quantity of the upstream in Xinjiang; finally, Kazakhstan is in general a net exporter of virtual water, so it lacks more water resources, and forcing it to pay more attention to the water quantity of the Ili River.

#### 5.2.2. A Brief Analysis on Strategies to Reduce Water Conflict with Virtual Water Transfer

Several strategies are proposed to reduce water conflict with virtual water transfer. First, a fair and reasonable plan to allocate water rights is critical in solving water quantity conflict [2,75,76]. The allocation plan must consider both physical and virtual water. Moreover, integrating virtual water included in trade into the water rights allocation, and the economic interests of both countries must be taken into account, to accurately measure the inequity of virtual water transfer. Then, based on virtual water transfer inequity and water stress, the amount of water allocated to each basin country is determined.

The brief management strategy is as follows: WSI in the riparian zones of Ili River in China and Kazakhstan reveals intermediate stress. Water stresses are relatively high, so making fair and reasonable allocation plans of water rights iskey to solving water quantity conflict. In addition, as a whole, China is in general a net importer of virtual water, and Kazakhstan is in general a net exporter of virtual water. Therefore, if China also gains the economic interests, it means China has an advantageous position. In order to decrease water quantity conflict of Ili River, China should moderately reduce its expectation of water allocation. If Kazakhstan gains the economic interests, we need to construct an inequity index to determine who has an advantageous position. Then, deciding who should decrease the psychological expectation of water allocation to reduce water quantity conflict. We will look into the detailed management strategy in our future research.

In addition, as a net exporter of virtual water, Kazakhstan needs to restructure its economy and trade, to reduce virtual water export and increase import. For that, Kazakhstan should accelerate intensive economy by developing water-saving and recycling technologies and upgrading structure of foreign trade to reduce water use intensity in various industries, especially agriculture, and restructure the investment and production of intermediate products.

## 6. Conclusions

Fresh water is remarkably scarce, accounting for just 2.5% of the global water supply—a valuable resource especially for semi-arid and arid countries [77]. Water conflicts are frequent for those countries due to the scarce freshwater. Freshwater includes physical water and virtual water. Virtual water is usually embedded in product and transferred during foreign trade. Most current studies about water conflict are centered on physical water and its impact on water quantity conflict, while fewer studies pay attention to the impact of virtual water transfer on water quantity conflict. This study considers the Ili River as an example, collects data on scarcity data on virtual water transfer from 1990 to 2015, calculates WSI under both scenarios (with or without virtual water transfer), and builds models to assess and predict water stress. Based on the LM Algorithm, CQs of the two scenarios are compared, and the quantitative relation between water stress and water conflict is constructed under both scenarios to explore the invisible effect of virtual water transfer on water conflict. Main conclusions are as follows:

(1) China was basically a net virtual water exporter from 1990 to 1995, and Kazakhstan a net exporter after 1995.
(2) Water stress in both countries along the Ili River increased in general.
(3) The impact of virtual water transfer from trade between the two countries on the total water availability after 1995 was more stable than the impact before 1995.
(4) Virtual water transfer had an invisible enhancing influence on the water conflict in Ili River.
(5) Exacerbated water stress in Kazakhstan is the main reason for the increased water conflict between the two countries in the riparian zone of Ili River.

## Figures and Tables

**Table 1:** Indicators to assess water stress without virtual water transfer.

Research Objective	Indicators		Variables (m^3^/Year)	Symbol
Water stress	Total annual water availability	Annual water availability (annual runoff)	Annual precipitation	*P*
			Annual evapotranspiration	*ET*
		Other water availability	Desalinated water	$Q_{3}$
			Imported physical water	$Q_{4}$
	Total annual water consumption		Agricultural use	$W_{1}$
			Industrial use	$W_{2}$
			Ecological use	$W_{3}$
			Domestic use	$W_{4}$

**Table 2:** Indicators to assess water stress with virtual water transfer.

Research Objective	Indicators		Variables (m^3^/Year)	Symbol
Water stress	Total annual water availability	Annual water availability(annual runoff)	Annual precipitation	P
			Annual evapotranspiration	ET
		Other water availability	Desalinated water	$Q_{3}$
			imported physical water	$Q_{4}$
			Virtual Water Transfer	$Q_{5}$
	Total annual water consumption		Agricultural use	$W_{1}$
			Industrial use	$W_{2}$
			Ecological use	$W_{3}$
			Domestic use	$W_{4}$

**Table 3:** WSI of the study area without virtual water transfer.

Year	WSI in Ili River Basin of China without Virtual Water Transfer	WSI in Ili River Basin of Kazakhstan without Virtual Water Transfer
1990–1995	0.288	0.214
1995–2000	0.249	0.204
2000–2005	0.273	0.247
2005–2010	0.445	0.446
2010–2015	0.402	0.421
2020–2025	0.426	0.429

**Table 4:** WSI of the study area with virtual water transfer.

Year	WSI in China	WSI in Kazakhstan
1990–1995	0.335	0.197
1995–2000	0.248	0.205
2000–2005	0.271	0.248
2005–2010	0.438	0.451
2010–2015	0.401	0.428
2020–2025	0.425	0.433

## Appendix A

**Determining the indicators of water stress:**

As the annual water availability (P−ET), agricultural ($W_{1}$), industrial ($W_{2}$), ecological ($W_{3}$), and domestic ($W_{4}$) water use needs to be calculated. This section focuses on determining the estimates of these indicators. Moreover, the data of P,ET come from NASA [64]. The data of agricultural, industrial, and domestic areas come from ESA [65]. Using the riparian areas of country r as an example.

(1) Annual water availability

(A1) $$\;\;\;P^{r}-ET^{r}=(P^{ar}-ET^{ar})\times {\;S}^{r}\times 12$$

where $P^{ar}$ is the average annual precipitation (unit: millimetre), $ET^{ar}$ is the average annual evapotranspiration (unit: millimetre). $S^{r}$ is the riparian area in country *r*. In order to obtain the annual water, we need to multiply the riparian area, and multiply by 12.

(2) Total annual water consumption
  (i) Agricultural water use $\left(W_{1}^{r}\right)$:
    (A2) W1r=W1×SbrSb 
    where $S^{br}$ is the agricultural land area of the riparian areas in country *r*, and $S^{b}$ is the total area of agricultural land of the riparian areas.
  (ii) Industrial and domestic water use $\left(W_{2}^{r}+W_{4}^{r}\right)$. As built-up area includes the area of residential land and industrial land, therefore the amount of domestic and industrial water use in the riparian zone in country *r* can be measured from built-up area:
    (A3) W2r+W4r=W2+W4×ScrSc
    where $S^{cr}$ is the built-up area of the riparian areas in country *r*, and $S^{c}$ is the total area of built-up area of the riparian areas.
  (iii) Ecological water use ($W_{3}^{r}$). The data of ecological water use is difficult to obtain. According to Mekonnen and Hoekstra [49], if ecological water use is less than 20% of the available water, it will pose a threat to the river ecosystem. Therefore, it is assumed that ecological water use in the riparian zone of each country is 20% of the available water.

(A4)
$$W_{3}^{r}=20\%\times \left(P^{r}-ET^{r}\right)$$

**Quantifying water quantity conflict**

The TFDD database is mainly based on the number of water conflicts (CN) and water conflict level (CL) to determine the severity of water quantity conflict, but did not combine them [58]. Gunasekara et al. [13] quantified water conflict based on the number of water conflicts: water conflict was high if there were water conflicts at least three or more years within a five-year period; water conflict was low if there were water conflicts only one or two years within a five-year period; no water conflict if there is no water conflict problem for 5 years. While, the quantification of water conflicts was limited to the number of conflicts. Therefore, in order to quantify water quantity conflict, this paper first defines the number of water conflicts (CN) and water conflict level (CL). Moreover, given that either CL or CN does not fully reveal the intensity of conflict, two variables—CN and CL—are chosen to measure the intensity of water quantity conflict (CQ).

(1) CN

According to Gunasekara N K et al. [13]: if, within a year (from 1 January to 31 December), countries within a drainage basin agree on water use, or express no dissatisfaction verbally or take no hostile or military actions, then they have no water conflicts, which is marked as 0. Otherwise, it is assumed that water quantity conflict occurs between the countries, which is marked as 1 [13]. The detailed expression is:

(A5) $$\mathrm{CN}=\left\{\begin{array}{l} 0,\;No\;water\;quantity\;conflict\;\\ 1,\;\;Water\;quantity\;conflict \end{array}\right.\;$$

In addition, to get an accurate result, this study sets a five-year timetable of the number of water conflict (CN) for statistics.

(2) CL

According to the Freshwater Resource Dispute Database (TFDD) at Orogan State University, USA [66]. CL is determined and quantified as follows:

(A6) $$\mathrm{CL}=\left\{\begin{array}{c} -1,\;\mathrm{mild}\;\mathrm{verbal}\;\mathrm{expressions}\;\mathrm{displaying}\;\mathrm{discord}\;\mathrm{in}\;\mathrm{interaction}\\ -2,\mathrm{strong}\;\mathrm{verbal}\;\mathrm{expressions}\;\mathrm{displaying}\;\mathrm{hostility}\;\mathrm{in}\;\mathrm{interaction}\\ -3,\;\mathrm{Diplomatic}-\mathrm{economic}\;\mathrm{hostile}\;\mathrm{actions}\\ -4,\;\mathrm{Political}-\mathrm{military}\;\mathrm{hostile}\;\mathrm{actions}\\ -5,\;\mathrm{Small}\;\mathrm{scale}\;\mathrm{military}\;\mathrm{acts}\\ -6,\;\mathrm{Extensive}\;\mathrm{War}\;\mathrm{Acts}\;\mathrm{causing}\;\mathrm{deaths},\;\mathrm{dislocation}\;\mathrm{or}\;\mathrm{high}\;\mathrm{strategic}\;\cos t\;\\ -7,\;\mathrm{Formal}\;\mathrm{Declaration}\;\mathrm{of}\;\mathrm{War} \end{array}\right.$$

Then, within a year (from 1 January to 31 December), after water conflict occurs (CN = 1), the countries within the same drainage basin that express dissatisfaction or take hostile actions are counted to determine CL. Similarly, we set a five-year timetable of CL for statistics.

(3) Determining CQ according to CN and CL

CQ is determined based on CN over a five-year period and the corresponding CL after each conflict occurs. CN and CL are combined to obtain CQ of transboundary rivers:

(A7) $$\mathrm{CQ}=w_{1}\mathrm{CN}+w_{2}\left|\mathrm{CL}\right|$$

where $w_{1}$ and $w_{2}$ are the weights of CN and CL respectively. TFDD grades water conflict as Level 1 to Level 7, and the absolute value of CL is selected for calculation. Next, based on the predicted WSI with or without virtual water transfer, the predicted CQ in the next five-year period can be figured out respectively.

(4) Determining the weights

According to TFDD, we first list all transboundary rivers involved in two or more water conflicts since 1948 (see Table A2). The number and accumulated levels of water quantity conflicts are then counted, and normalized via linear transformation, so as to determine the weights of CN and CL through the entropy weighting method.

**The entropy weighting method to determine the weights of CN and CL**

**Formula derivation process:**

① Water Stress Indicator normalization process

Since the number of water conflicts (CN) and water conflict level (CL) have a positive effect on water quantity conflict, this paper selects a positive indicator treatment to normalize the actual number of conflicts and cumulative conflict levels in Table A1. We get:

(A8) ∂ij=xij−mini=1,…,25xijmaxi=1,…,25xij−mini=1,…,25xij

where i=1,…,25, j=1,2; $\partial_{ij}\;$ is the actual number of water quantity conflicts and the cumulative conflict level normalized for each transboundary river; $\underset{i=1,\ldots ,25}{\min}\left\{x_{ij}\right\}$ is the minimum value in column *j*, and $\underset{i=1,\ldots ,25}{\max}\left\{x_{ij}\right\}$ is the maximum value in column *j*.

② Entropy weighting method to determine the weights

The entropy weight method has higher accuracy and objectivity compared to the subjective weight determination method. Since CN and CL have objective metrics, the entropy weighting method is selected to determine the two weights in this paper. The details are as follows:

First determine the information entropy of CN and CL:

(A9) $$E_{j}=\left(-ln{\left(n\right)}^{-1}\right)\sum_{i=1}^{n}\gamma_{ij}ln\gamma_{ij}$$

where γij=∂ij∑i=1n∂ij, if $\gamma_{ij}=0$, then define $\underset{\gamma_{ij}\to 0}{\lim}\overset{n}{\underset{i=1}{\sum }}\gamma_{ij}ln\gamma_{ij}=0$. *n* is the number of transborder rivers, i.e., *n* = 25.

Second, the weights of the two indicators are determined based on the information entropy of CN and CL:

(A10) wj=1−Ej∑j=121−Ej

**The specific solution procedure of Levenberg–Marquardt (LM) Algorithm:**

Let the variables $x_{1},\;x_{2},\cdots ,x_{m-1}$ and CQ have *M* − 1 sets of observations and obtain *M* − 1 sets of data ($x_{K1},\;x_{K2},\cdots ,x_{K\left(m-1\right)},{\mathrm{CQ}}_{K}$), respectively, where K=1,2,⋯,M−1. Bringing in $X_{K}$ and the corresponding function values, we can get:

(A11) $$f\left(x_{K1},\;x_{K2},\cdots ,x_{K\left(m-1\right)};d_{1},\;d_{2},\cdots ,d_{p}\right)=f\left(x_{K};d\right)$$

Assigning initial values $\;d^{\left(0\right)}=d_{1}^{\left(0\right)},\;d_{2}^{\left(0\right)},\cdots ,d_{p}^{\left(0\right)}\;$ to the d values in each group of observations. For a given initial value $d_{i}^{\left(0\right)}$, we have $d_{i}-d_{i}^{\left(0\right)}=\Delta_{i}\left(i=1,2,\cdots p\right)$.

Expanding $f\left(x_{K};d\right)$ at the initial value $d^{\left(0\right)}\;$ with Taylor series, and retaining only the constant and primary terms of the expansion, we obtain:

(A12) $$f\left(x_{K};d\right)\approx f\left(x_{K};d^{\left(0\right)}\right)+\sum_{i=1}^{p}{\left.\frac{\partial f\left(x_{K};d\right)}{\partial d_{i}}\right|}_{d=d^{\left(0\right)}}\left(d_{i}-d_{i}^{\left(0\right)}\right)\;$$

Equation (A5) can be viewed as a linear functional form with respect to the parameters $d_{1},\;d_{2},\cdots ,d_{p}$, and the residual sum of squares Q is calculated for *M* sets of data according to the least squares method.

(A13) $$Q=\overset{M-1}{\underset{k=1}{\sum }}{\left[CQ_{K}-f\left(x_{K};d\right)\right]}^{2}\;\;\approx \overset{M-1}{\underset{k=1}{\sum }}{\left[CQ_{K}-f\left(x_{K};d^{\left(0\right)}\right)-\sum_{i=1}^{p}{\left.\frac{\partial f\left(x_{K};d\right)}{\partial d_{i}}\right|}_{d=d^{\left(0\right)}}\left(d_{i}-d_{i}^{\left(0\right)}\right)\right]}^{2}\;\;$$

The main idea of Gauss–Newton method is that given the initial values of parameters $d^{\left(0\right)}=d_{1}^{\left(0\right)},\;d_{2}^{\left(0\right)},\cdots ,d_{p}^{\left(0\right)}$, the iterative change of parameters $\;\Delta =d-d^{\left(0\right)}$ is solved, then $d_{i}-d_{i}^{\left(0\right)}=\Delta_{i}\left(i=1,2,\ldots p\right)$ until the parameters converge. The McQuart algorithm adds a correction term to the above equation, which becomes:

(A14) $$Q=\overset{M-1}{\underset{k=1}{\sum }}{\left[CQ_{K}-f\left(x_{K};d\right)\right]}^{2}\;\;\approx \overset{M-1}{\underset{k=1}{\sum }}{\left[CQ_{K}-f\left(x_{K};d^{\left(0\right)}\right)-\sum_{i=1}^{p}{\left.\frac{\partial f\left(x_{K};d\right)}{\partial d_{i}}\right|}_{d=d^{\left(0\right)}}\Delta_{i}\right]}^{2}+\varepsilon \overset{p}{\underset{i=1}{\sum }}\Delta_{i}{}^{2}\;$$

where, *ε* ≥ 0, and is called the damping factor. To make the function dependent variable $Q=Q_{min}$, it is necessary to find the first-order partial derivatives of its p parameters to be found and make the result equal to zero, i.e., $\frac{\partial Q}{\partial d_{j}}=0$. Expanding this expression, we can obtain:

(A15) $$\overset{M-1}{\underset{k=1}{\sum }}\left[CQ_{K}-f\left(x_{K};d^{\left(0\right)}\right)-\sum_{i=1}^{p}{\left.\frac{\partial f\left(x_{K};d\right)}{\partial d_{i}}\right|}_{d=d^{\left(0\right)}}\Delta_{i}\right]\left({\left.\frac{\partial f\left(x_{K};d\right)}{\partial d_{j}}\right|}_{d=d^{\left(0\right)}}\right)+\varepsilon \overset{p}{\underset{i=1}{\sum }}\Delta_{i}=0$$

In Equation (A8), *j* = 1, 2, ⋯, *p*, and translate Equation (A8) into the following form:

(A16) $$\left\{\begin{array}{c} \delta_{1CQ}=\left(\beta_{11}+\varepsilon \right)\Delta_{1}+\beta_{12}\Delta_{2}+\ldots +\beta_{1p}\Delta_{p}\\ \delta_{2CQ}=\beta_{21}\Delta_{1}+\left(\beta_{22}+\varepsilon \right)\Delta_{2}+\ldots +\beta_{2p}\Delta_{p}\\ \vdots \\ \delta_{pCQ}=\beta_{p1}\Delta_{1}+\beta_{p2}\Delta_{2}+\ldots +\left(\beta_{pp}+\varepsilon \right)\Delta_{p} \end{array}\right.$$

Equation (A9) can be transformed into:

(A17) $$\left|\begin{array}{c} \Delta_{1}\\ \Delta_{2}\\ \vdots \\ \Delta_{p} \end{array}\right|={\left|\begin{array}{cccc} \left(\beta_{11}+\varepsilon \right) & \beta_{12} & \cdots & \beta_{1p}\\ \beta_{21} & \left(\beta_{22}+\varepsilon \right) & \cdots & \beta_{2p}\\ \cdots & \cdots & \cdots & \cdots \\ \beta_{p1} & \beta_{p2} & \cdots & \left(\beta_{pp}+\varepsilon \right) \end{array}\right|}^{-1}\times \left|\begin{array}{c} \delta_{1CQ}\\ \delta_{2CQ}\\ \vdots \\ \delta_{pCQ} \end{array}\right|$$

where,

(A18) $$\beta_{ij}=\overset{M-1}{\underset{i=1}{\sum }}{\left.\frac{\partial f\left(x_{K};d\right)}{\partial d_{i}}\right|}_{d=d^{\left(0\right)}}\times {\left.\frac{\partial f\left(x_{K};d\right)}{\partial d_{j}}\right|}_{d=d^{\left(0\right)}},\left(i,j=1,\;2,\cdots ,p\;\right)$$

(A19) $$\delta_{iCQ}=\overset{M-1}{\underset{i=1}{\sum }}{\left.\frac{\partial f\left(x_{K};d\right)}{\partial d_{i}}\right|}_{d=d^{\left(0\right)}}\times \left(CQ_{K}-CQ_{K}^{\left(0\right)}\right)$$

It is known that the solution $d_{i}$ of the matrix is related to the initial values $d^{\left(0\right)}=d_{1}^{\left(0\right)},\;d_{2}^{\left(0\right)},\cdots ,d_{p}^{\left(0\right)}\;$ and the damping factor ε. When conducting the iteration, if the absolute value of $d_{i}-d_{i}^{\left(0\right)}=\Delta_{i}$ is smaller, the process of optimal solution of the parameters is finished; if it is larger, $d_{i}$, as the new initial value $d^{\left(0\right)}$, is substituted and recalculated. Follow this process iteratively until $\;\Delta_{i}$ is approximately negligible or no change, i.e., the result satisfies the requirement, and the process ends.

## Appendix B

**Table A1:** The modified multi-regional input–output table.

Output Input			Intermediate Use						Final Demand				Total Output
			Basin Country 1		Basin Country (m − 1)		Other Country m		Basin Country 1		BasinCountry (m − 1)	Other Country m	
			Industry 1	Industry n	Industry 1	Industry n	Industry 1	Industry n					
Intermediate use	Basin country 1	Industry 1	$y_{11}^{11}$	$y_{1n}^{11}$	$y_{11}^{1\left(m-1\right)}$	$y_{1n}^{1\left(m-1\right)}$	$y_{11}^{1m}$	$y_{1n}^{1m}$	$f_{1}^{11}$		$f_{1}^{1\left(m-1\right)}$	$f_{1}^{1m}$	$y_{1}^{1}$
		Industry n	$y_{n1}^{11}$	$y_{nn}^{11}$	$y_{n1}^{1\left(m-1\right)}$	$y_{nn}^{1\left(m-1\right)}$	$y_{n1}^{1m}$	$y_{nn}^{1m}$	$f_{n}^{11}$		$f_{n}^{1\left(m-1\right)}$	$f_{n}^{1m}$	$y_{n}^{1}$
	Basin country (m − 1)	Industry 1	$y_{11}^{\left(m-1\right)1}$	$y_{1n}^{\left(m-1\right)1}$	$y_{11}^{\left(m-1\right)\left(m-1\right)}$	$y_{1n}^{\left(m-1\right)\left(m-1\right)}$	$y_{11}^{\left(m-1\right)m}$	$y_{1n}^{\left(m-1\right)m}$	$f_{1}^{\left(m-1\right)1}$		$f_{1}^{\left(m-1\right)\left(m-1\right)}$	$f_{1}^{1m}$	$y_{1}^{m-1}$
		Industry n	$y_{n1}^{\left(m-1\right)1}$	$y_{nn}^{\left(m-1\right)1}$	$y_{n1}^{\left(m-1\right)\left(m-1\right)}$	$y_{nn}^{\left(m-1\right)\left(m-1\right)}$	$y_{n1}^{\left(m-1\right)m}$	$y_{nn}^{\left(m-1\right)m}$	$f_{n}^{\left(m-1\right)1}$		$f_{n}^{\left(m-1\right)\left(m-1\right)}$	$f_{n}^{1m}$	$y_{n}^{m-1}$
	Other country m	Industry 1	$y_{11}^{m1}$	$y_{1n}^{m1}$			$y_{11}^{mm}$	$y_{1n}^{mm}$	$f_{1}^{m1}$		$f_{1}^{m\left(m-1\right)}$	$f_{1}^{mm}$	$y_{1}^{m}$
		Industry n	$y_{n1}^{m1}$	$y_{nn}^{m1}$			$y_{n1}^{mm}$	$y_{nn}^{mm}$	$f_{n}^{m1}$	$f_{n}^{m2}$	$f_{n}^{m\left(m-1\right)}$	$f_{n}^{mm}$	$y_{n}^{m}$
Added Value			$v_{1}^{1}$	$v_{n}^{1}$			$v_{1}^{m}$	$v_{n}^{m}$					
Total input			$y_{1}^{1}$	$y_{n}^{1}$			$y_{1}^{m}$	$y_{n}^{m}$					
Direct water input			$w_{n}^{r}$										

**Table A2:** The actual number and grade of water quantity conflicts in transboundary rivers.

Transboundary River Names	The Actual Number of Water Quantity Conflicts	The Cumulative Conflict Rank	Transboundary River Names	The Actual Number of Water Quantity Conflicts	The Cumulative Conflict Rank
Nile	20	−43	St. Lawrence	4	−6
Rio Grande	12	−20	Asi/Orontes	7	−8
Colorado	11	−16	Muhuri	2	−2
Indus	24	−37	Douro	3	−3
Ganges	46	−71	Mino	3	−3
Helmand	7	−8	Vardar	4	−7
Aral sea	4	−14	Lauca/cancoso	4	−5
Tigris-Euphrates	101	−151	Karnafauli	2	−2
Jordan	160	−373	Mekong	5	−5
Guadiana	5	−5	Ob	2	−4
Senegal	5	−11	Danube	7	−13
La Plata	4	−7	Tagus/Tejo river	3	−3
Nelson	4	−4			
